# Computational Analysis of topological indices of two Boron Nanotubes

**DOI:** 10.1038/s41598-018-33081-y

**Published:** 2018-10-04

**Authors:** Young Chel Kwun, Mobeen Munir, Waqas Nazeer, Shazia Rafique, Shin Min Kang

**Affiliations:** 1 Department of Mathematics, Dong-A University, Busan, 49315 Korea; 2 Division of Science and Technology, University of Education, Lahore, 54000 Pakistan; 3 Center for Excellence in Molecular Biology, Punjab University Lahore, Lahore, 53700 Pakistan; 4 Department of Mathematics and RINS, Gyeongsang National University, Jinju, 52828 Korea; 5 Center for General Education, China Medical University, Taiwan, Taichung 40402 Taiwan

## Abstract

There has been a recent debate that boron nanotubes can outperform carbon nanotubes on many grounds. The most stable boron nanotubes are made of a hexagonal lattice with an extra atom added to some of the hexagons called ∝-boron nanotubes. Closed forms of M-polynomial of nanotubes produce closed forms of many degree-based topological indices which are numerical parameters of the structure and determine physico-chemical properties of the concerned nanotubes. In this article, we compute and analyze many topological indices of ∝-boron nanotubes correlating with the size of structure of these tubes through the use of M-polynomial. More importantly we make a graph-theoretic comparison of indices of two types of boron nanotubes namely triangular boron and ∝-boron nanotubes.

## Introduction

Mathematical chemistry provides tools such as polynomials and functions to capture information hidden in the symmetry of molecular graphs and thus predict properties of compounds without using quantum mechanics. A topological index isa numerical parameter of a graph and depicts its topology. It describes the structure of molecules numerically and are used in the development of qualitative structure activity relationships (QSARs). Most commonly known invariants of such kinds are degree-based topological indices. These are actually the numerical values that correlate the structure with various physical properties, chemical reactivity and biological activities^1–5^. It is an established fact that many properties such as heat of formation, boiling point, strain energy, rigidity and fracture toughness of a molecule are strongly connected to its graphical structure.

Hosoya polynomial, (Wiener polynomial)^6^, plays a pivotal role in distance-based topological indices. A long list of distance-based indices can be easily evaluated from Hosoya polynomial. A similar breakthrough was obtained recently by Klavzar *et*. *al*.^7^, in the context of degree-based indices. Authors in^7^ introduced M-polynomial in, 2015, to play a role, parallel to Hosoya polynomial to determine closed form of many degree-based topological indices^8–12^. The real power of M-polynomial is its comprehensive nature containing healthy information about degree-based graph invariants. These invariants are calculated on the basis of symmetries present in the 2d-molecular lattices and collectively determine some properties of the material under observation.

**Figure 1:**
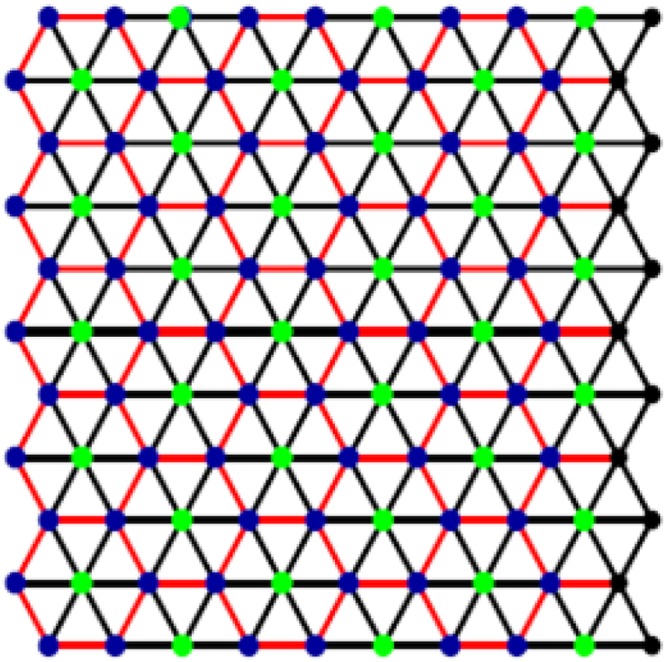
Triagular Boron Tube.

Because of increasing interests and developments of new nanomaterials, computations have minimized the burden of experimental labor to some extent. Amongst the nanomaterials, nanocrystals, nanowires and nanotubes, constitute three major categories, the last two being one-dimensional. Boron nanotubes are becoming increasingly interesting because of their remarkable properties like structural stability, work function, transport properties, and electronic structure^13^. Triangular Boron is derived from a triangular sheet as shown in Fig. 1. The first boron nanotubes were created, in 2004, from a buckled triangular latticework^13–15^.

**Figure 2:**
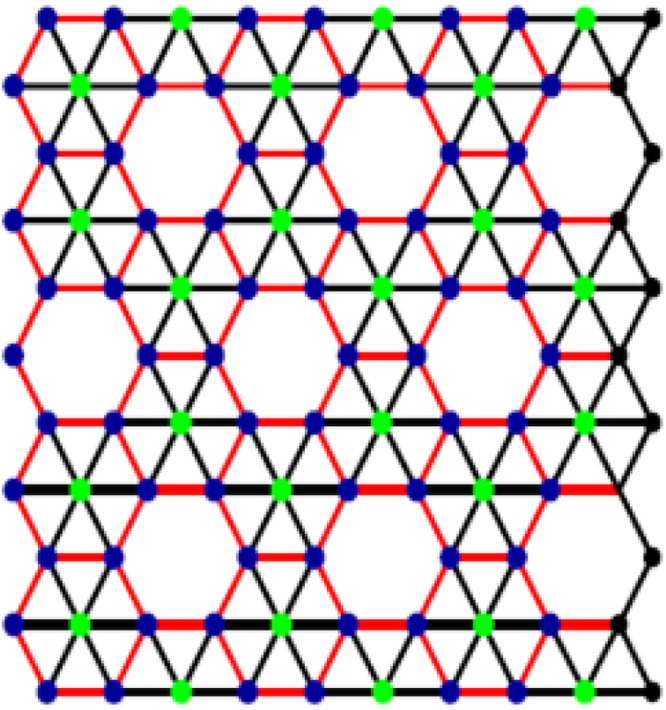
∝- Boron nanotube.

**Figure 3:**
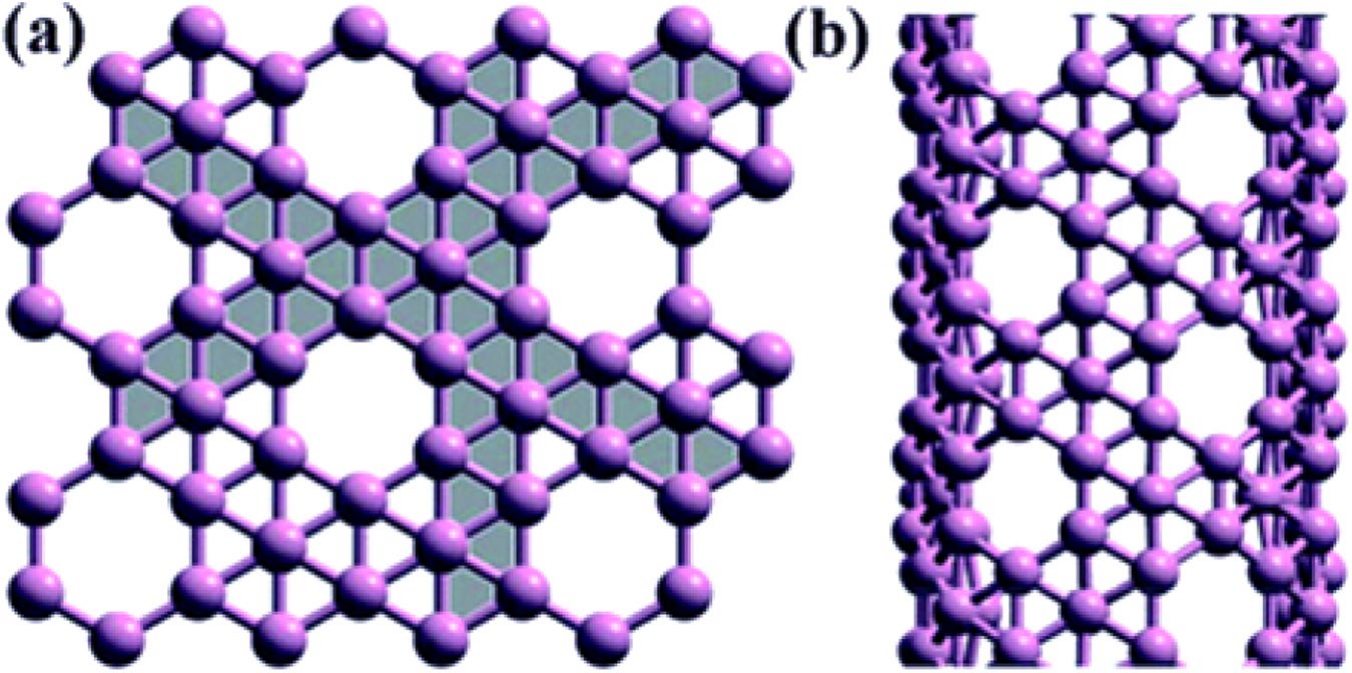
The molecular structure of ∝- boron nanotube.

These tubes are discussed recently in^15,16^. Other well-known type, α-boron, is derived from α-sheet. Irrespective of their structures and chiralities, both types are more conductive than carbon nanotubes^14,17–19^. Figure 2 describes basic structure of ∝- boron nanotube. Following Fig. 3 also presents different views of ∝- Boron nanotube, (a) is the planar view whereas (b) is the tabular view.

As for as structure of both tubes are concerned, ∝- Boron nanotube is more complicated than Triangular boron nanotubes with addition of an extra atom to the center of some of the hexagons^15^. In^15^, authors proved that this is the most stable known theoretical structure for a boron nanotube. They also showed that, with this pattern, boron nanotubes should have variable electrical properties: wider ones would be metallic conductors, but narrower ones should be semiconductors. So, these tubes boron tubes will be used in Nano devices similar to the diodes and transistors that have already been made from carbon nanotube. In^16^ authors computed some computational facts which are similar in both types of boron nanotubes and carbon nanotubes. Munir *et al*. computed M-polynomial and related indices of triangular boron nanotubes in^11^, polyhex nanotubes in^12^, nanostar dendrimers in^8^, titania nanotubes in^9^ and M- Polynomials and topological indices of V-Phenylenic Nanotubes and Nanotori in^10^. In all above mentioned articles we presented an analysis of these indices against the parameters of structure involved. In this article, we compute general form of M-polynomial for ∝- boron nanotube. Then we derive closed forms of many degree-based topological indices for these tubes. We also draw some conclusions about both types of boron tubes.

## Basic Definitions and Literature Review

Throughout this article, we assume G to be a connected graph, *V* (*G*) and *E* (*G*) are the vertex set and the edge set respectively and *d*_*v*_ denotes the degree of a vertex *v*.

### Definition 1

. The M-polynomial of G is defined as: $M(G,x,y)=\sum _{\delta \le i\le j\le \Delta }{m}_{ij}(G){x}^{i}{y}^{j}$ where *δ* = Min{*d*_*v*_|*v* ∈ V (G)}, Δ = Max{*d*_*v*_|*v* ∈ V (G)}, and *m*_*ij*_(*G*) is the edge *vu* ∈ *E*(*G*) such that where ≤*j*^7^.

Wiener index and its various applications are discussed in^20–22^. Randić index, *R*_−1/2_(*G*), is introduced by Milan Randić in 1975 defined as: ${R}_{-1/2}(G)=\sum _{uv\in E(G)}\frac{1}{\sqrt{{d}_{u}{d}_{v}}}.$ For general detains about *R*_−1/2_(*G*) and its generalized Randić index, ${R}_{\alpha }(G)=\sum _{uv\in E(G)}\frac{1}{{({d}_{u}{d}_{v})}^{\alpha }},$ please see^23–28^ and the inverse Randić index is defined as $R{R}_{\alpha }(G)=\sum _{uv\in E(G)}{({d}_{u}{d}_{v})}^{\alpha }.$ Clearly *R*_−1/2_(*G*) is a special case of *R*_*α*_(*G*) when $\alpha =-\,\frac{1}{2}$. This index has many applications in diverse areas. Many papers and books such as^29–31^ are written on this topological index as well. Gutman and Trinajstić introduced two indices defined as: ${M}_{1}(G)=\sum _{uv\in E(G)}({d}_{u}+{d}_{v})$ and ${M}_{2}(G)=\sum _{uv\in E(G)}({d}_{u}\times {d}_{v})$. Thesecond modified Zagreb index is defined as: ${}^{m}M_{2}(G)=\sum _{uv\in E(G)}\frac{1}{d(u)d(v)}.$ We refer^32–36^ to the readers for comprehensive details of these indices. Other famous indices are Symmetric division index: ${\rm{SDD}}({\rm{G}})=\sum _{uv\in E(G)}\{\frac{{\rm{\min }}({d}_{u},{d}_{v})}{{\rm{\max }}({d}_{u},{d}_{v})}+\frac{{\rm{\max }}({d}_{u},{d}_{v})}{{\rm{\min }}({d}_{u},{d}_{v})}\}$ harmonic index defined $H(G)=\sum _{vu\in E(G)}\frac{2}{{d}_{u}+{d}_{v}}.$ Inverse sum index i: $I(G)=\sum _{vu\in E(G)}\frac{{d}_{u}{d}_{v}}{{d}_{u}+{d}_{v}}$ and augmented Zagreb index: $A(G)=\sum _{vu\in E(G)}{\{\frac{{d}_{u}{d}_{v}}{{d}_{u}+{d}_{v}-2}\}}^{3},$^37,38^.

Tables presented in^7–11^ relates some of these well-known degree-based topological indices with M-polynomial with following reserved notations

1 $$\begin{array}{rcl}{D}_{x} & = & x\frac{\partial (f(x,y)}{\partial x},{D}_{y}=y\frac{\partial (f(x,y)}{\partial y},\,\\ {S}_{x} & = & {\int }_{0}^{x}\frac{f(t,y)}{t}dt,{S}_{y}={\int }_{0}^{y}\frac{f(x,t)}{t}dt,\,\\ J(f(x,y)) & = & f(x,x),{Q}_{\alpha }(f(x,y))={x}^{\alpha }f(x,y).\end{array}$$

## Computational Results

**Figure 4:**
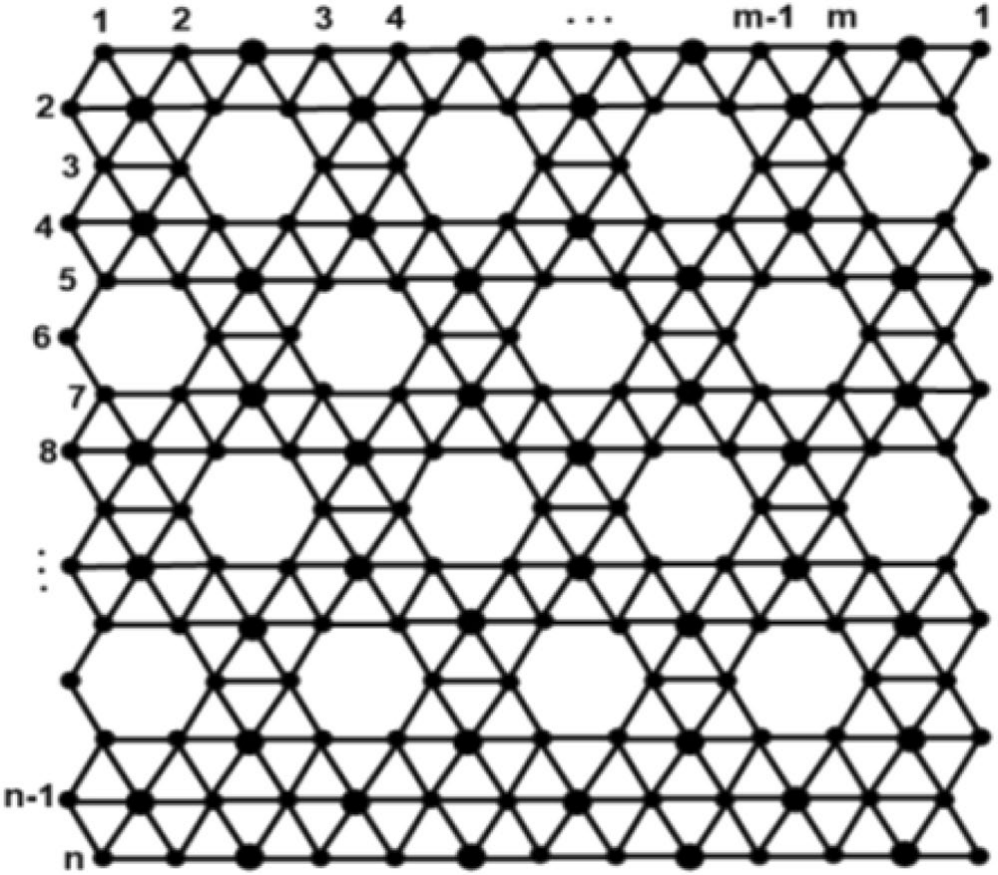
2D-lattice structure of ∝- boron nanotube.

In this section, we give our computational results. In terms of chemical graph theory and mathematical chemistry, we associate a graph with the molecular structure where vertices correspond to atoms and edges to bonds. Following the same lines, we represent a ∝- boron nanotube, by a planar graph, *BNT*_*t*_[*m*, *n*] of order n × m, as the in Fig. 3 demonstrates. Clearly, from Fig. 4, there are $\frac{3nm}{2}$ vertices and $\frac{3}{2}mn+3{m}^{2}+\frac{9}{2}m+n-3$ edges in 2D graph model of ∝-boron nanotubes.

### **Theorem 1**.

Let *BNT*_*α*_[*m*, *n*] is ∝-boron Nanotubes. Then

2 $$\begin{array}{rcl}M(BN{T}_{\alpha }[m,n],x,y) & = & 3m{x}^{4}{y}^{4}+2m{x}^{4}{y}^{5}+4m{x}^{4}{y}^{6}\\  &  & +\frac{m}{2}(3n-11){x}^{5}{y}^{5}+3{m}^{2}{x}^{5}{y}^{6}+(m+n-3){x}^{6}{y}^{6}.\end{array}$$

### ***Proof***.

Let *BNT*_*α*_[*m*, *n*] be ∝-boron nanotubes, where m is the number of columns and n is the number of rows. From Fig. 4 it is easy to observe that the graph of *BNT*_*α*_[*m*, *n*] has $\frac{4nm}{3}$ number of vertices and $\frac{3}{2}mn+3{m}^{2}+\frac{9}{2}m+n-3$ edges where *n* is multiple of 3.

The edge set of *BNT*_*α*_[*m*, *n*] has following three partitions,

3 $${V}_{\{4\}}=\{v{\grave{o}}BN{T}_{\alpha }[m,n]|{d}_{v}=4\},$$

4 $${V}_{\{5\}}=\{v{\grave{o}}BN{T}_{\alpha }[m,n]|{d}_{v}=5\}$$

And

5 $${V}_{\{6\}}=\{v{\grave{o}}BN{T}_{\alpha }[m,n]|{d}_{v}=6\},$$

and the vertex set *V*(*BNT*_*α*_[*m*, *n*]) of *BNT*_*α*_[*m*, *n*] has six partitions:

6 $${E}_{\{4,4\}}=\{e=uv\in E(BN{T}_{\alpha }[m,n])|{d}_{u}=4\,,\,{d}_{v}=4\},$$

7 $${E}_{\{4,5\}}=\{e=uv\in E(BN{T}_{\alpha }[m,n])|{d}_{u}=4\,,\,{d}_{v}=5\},$$

8 $${E}_{\{4,6\}}=\{e=uv\in E(BN{T}_{\alpha }[m,n])|{d}_{u}=4\,,\,{d}_{v}=6\},$$

9 $${E}_{\{5,5\}}=\{e=uv\in E(BN{T}_{\alpha }[m,n])|{d}_{u}=5\,,\,{d}_{v}=5\},$$

10 $${E}_{\{5,6\}}=\{e=uv\in E(BN{T}_{\alpha }[m,n])|{d}_{u}=5\,,\,{d}_{v}=6\},$$

11 $${E}_{\{6,6\}}=\{e=uv\in E(BN{T}_{\alpha }[m,n])|{d}_{u}=6\,,\,{d}_{v}=6\}.$$

Now

12 $$|{E}_{\{4,4\}}|=3m,$$

13 $$|{E}_{\{4,5\}}|=2m,$$

14 $$|{E}_{\{4,6\}}|=4m,$$

15 $$|{E}_{\{5,5\}}|=\frac{m}{2}(3n-11),$$

16 $$|{E}_{\{5,6\}}|=3{m}^{2},$$

and

17 $$|{E}_{\{6,6\}}|=m+n-3.$$

Thus the M-polynomial of *BNTα*[*m*, *n*] is:

18 $$\begin{array}{rcl}M(BN{T}_{\alpha }[m,n],\,x\,,y) & = & \sum _{i\le j}{m}_{ij}(BN{T}_{\alpha }[m,n]){x}^{i}{y}^{j}\\  & = & \sum _{4\le 4}{m}_{44}(BN{T}_{\alpha }[m,n]){x}^{4}{y}^{4}+\,\sum _{4\le 5}{m}_{45}(BN{T}_{\alpha }[m,n]){x}^{4}{y}^{5}\\  &  & +\,\sum _{4\le 6}{m}_{46}(BN{T}_{\alpha }[m,n]){x}^{4}{y}^{6}+\,\sum _{5\le 5}{m}_{55}(BN{T}_{\alpha }[m,n]){x}^{5}{y}^{5}\\  &  & +\,\sum _{5\le 6}{m}_{56}(BN{T}_{\alpha }[m,n]){x}^{5}{y}^{6}+\,\sum _{6\le 6}{m}_{66}(BN{T}_{\alpha }[m,n]){x}^{6}{y}^{6}\\  & = & \sum _{uv\in {E}_{\{4,4\}}}{m}_{44}(BN{T}_{\alpha }[m,n]){x}^{4}{y}^{4}+\,\sum _{uv\in {E}_{\{4,5\}}}{m}_{45}(BN{T}_{\alpha }[m,n]){x}^{4}{y}^{5}\\  &  & +\,\sum _{uv\in {E}_{\{4,6\}}}{m}_{46}(BN{T}_{\alpha }[m,n]){x}^{4}{y}^{6}\times \,\sum _{uv\in {E}_{\{5,5\}}}{m}_{55}(BN{T}_{\alpha }[m,n]){x}^{5}{y}^{5}\\  &  & +\,\sum _{uv\in {E}_{\{5,6\}}}{m}_{56}(BN{T}_{\alpha }[m,n]){x}^{5}{y}^{6}+\,\sum _{uv\in {E}_{\{6,6\}}}{m}_{66}(BN{T}_{\alpha }[m,n]){x}^{6}{y}^{6}\\  & = & |{E}_{\{4,4\}}|{x}^{4}{y}^{4}+|{E}_{\{4,5\}}|{x}^{4}{y}^{5}+|{E}_{\{4,6\}}|{x}^{4}{y}^{6}+\,|{E}_{\{5,5\}}|{x}^{5}{y}^{5}+|{E}_{\{5,6\}}|{x}^{5}{y}^{6}+|{E}_{\{6,6\}}|{x}^{6}{y}^{6}\\  & = & 3m{x}^{4}{y}^{4}+2m{x}^{4}{y}^{5}+4m{x}^{4}{y}^{6}+\frac{m}{2}(3n-11){x}^{5}{y}^{5}+\,3{m}^{2}{x}^{5}{y}^{6}+(m+n-3){x}^{6}{y}^{6}.\end{array}$$

**Figure 5:**
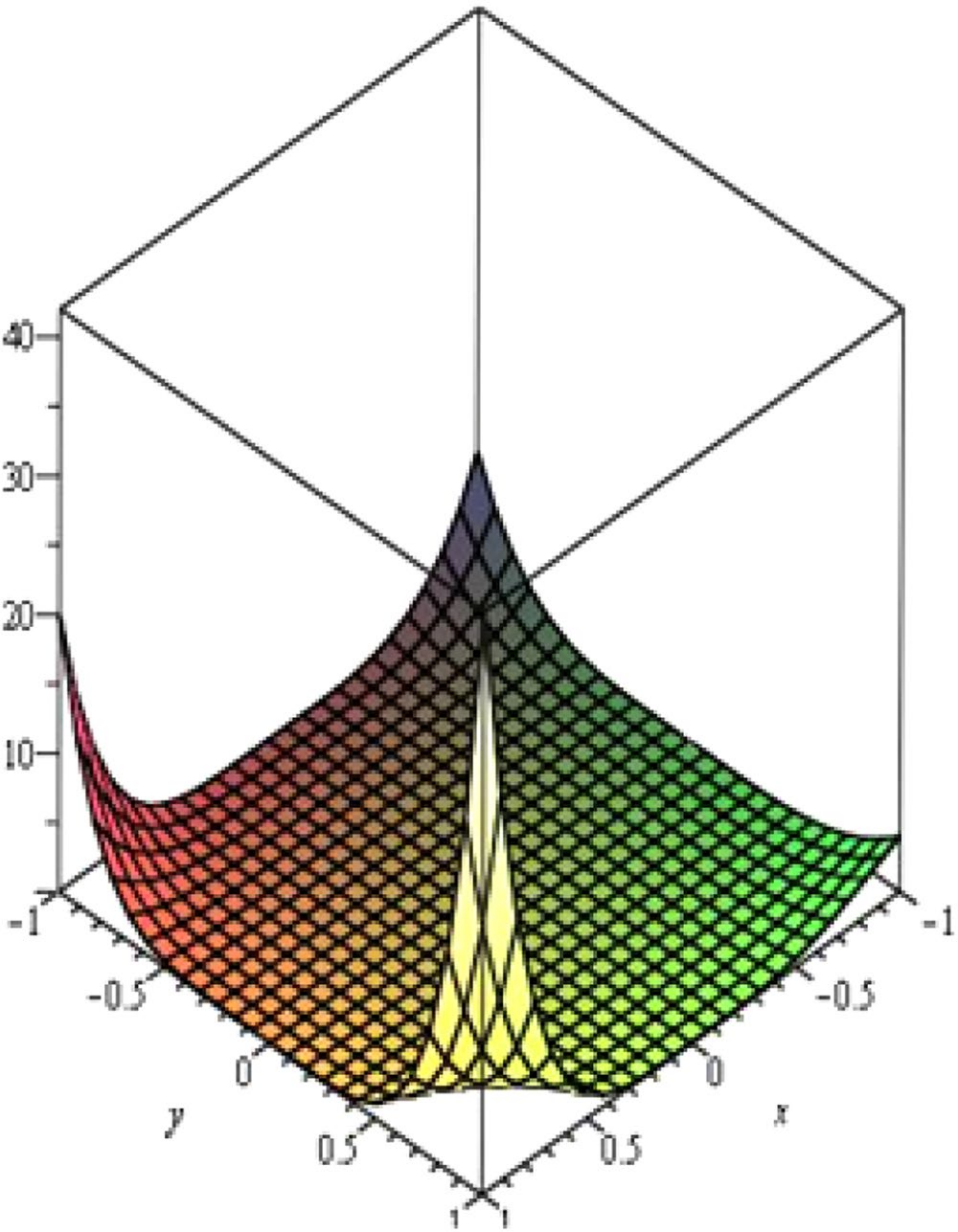
Plot of M-polynomial of *BNT*_*α*_[*m*, *n*].

**Figure 6:**
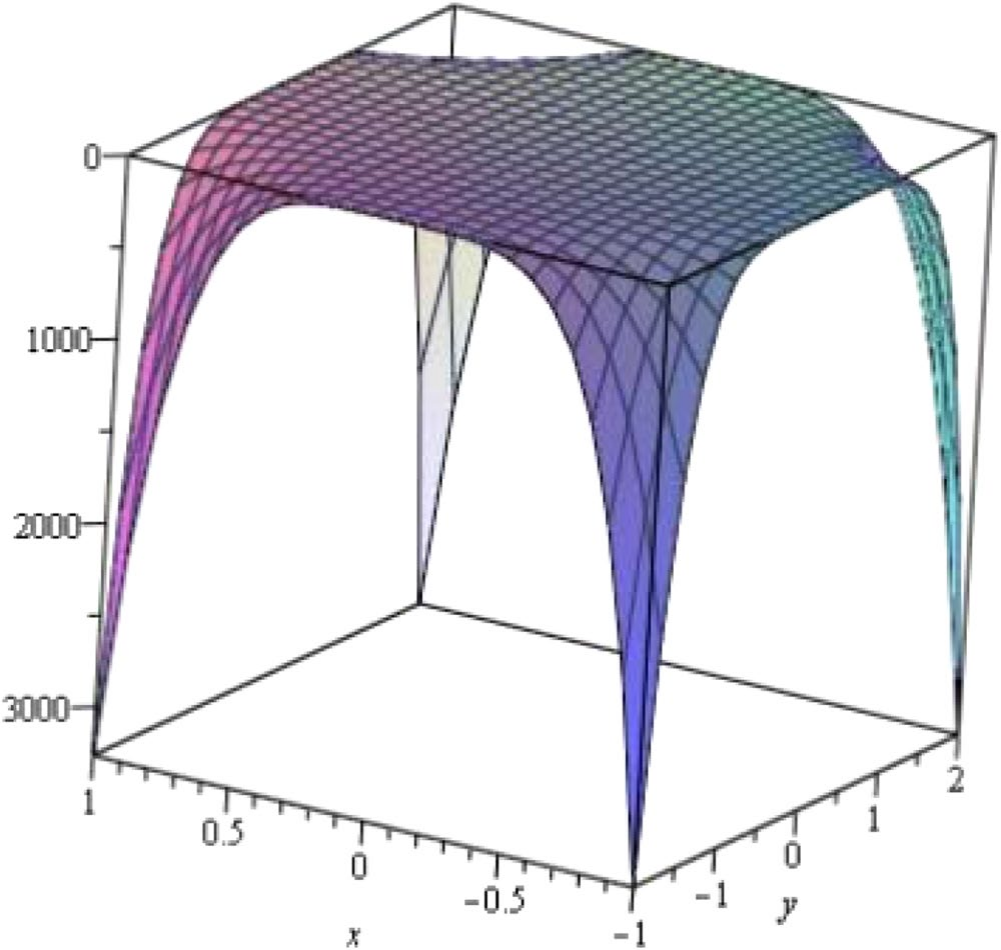
3D plot of the M-polynomial of Triangular boron Tube.

Above Fig. 5 presents the Maple 13 plot of the M-polynomial of ∝- Boron Nanotubes. Clearly, values drastically increases as Χ → ±1, *Y* → ±2 For the most part of [−1, 1] × [−2, 2], values remain stable whereas triangular boron nanotubes show opposite trends^11^, shown in Fig. 6 below

Now we derive formulas for many degree-based topological indices using M-polynomial.

### **Proposition 1**

Let *BNT*_*α*_[*m*, *n*] is the boron ∝- nanotube, then

1. $${M}_{1}(G)=94m+5m(3n-11)+33{m}^{2}+12n-36.$$
2. $${M}_{2}(G)=220m+\frac{25}{2}m(3n-11)+90{m}^{2}+36n-108.$$
3. $${}^{m}M_{2}(G)=\frac{1}{10}{m}^{2}+(\frac{943}{3600}+\frac{3}{50}n)m+\frac{1}{36}n-\frac{1}{12}.$$
4. $${R}_{\alpha }(G)=3\times {16}^{\alpha }m+2m{20}^{\alpha }+4m{24}^{\alpha }+\frac{1}{2}{25}^{\alpha }m(3n-11)+3{m}^{2}{30}^{\alpha }+{36}^{\alpha }(m+n-3).$$
5. $${R}_{\alpha }(G)=\frac{3}{{16}^{\alpha }}m+\frac{2}{{20}^{\alpha }}m+\frac{4}{{24}^{\alpha }}m+\frac{1}{2\times {25}^{\alpha }}m(3n-11)+\frac{3}{{30}^{\alpha }}{m}^{2}+\frac{1}{{36}^{\alpha }}(m+n-3).$$
6. $$SSD(G)=\frac{623}{30}m+m(3n-11)+\frac{61}{10}{m}^{2}+2n-6.$$
7. $$H(G)=\frac{6}{11}{m}^{2}+(\frac{191}{180}+\frac{3}{10}n)m+\frac{1}{6}n-\frac{1}{2}.$$
8. $$I(G)=\frac{90}{11}{m}^{2}+(\frac{1673}{180}+\frac{15}{4}n)m+3n-9.$$
9. $$A(G)=\frac{1000}{9}{m}^{2}+(\frac{35698735591}{395136000}+\frac{46875}{1024}n)m+\frac{5832}{125}n-\frac{17496}{125}.$$

### **Proof**.

Let

19 $$\begin{array}{rcl}M(G;x,y) & = & f(x,y)=3m{x}^{4}{y}^{4}+2m{x}^{4}{y}^{5}+4m{x}^{4}{y}^{6}\\  &  & +\frac{m}{2}(3n-11){x}^{5}{y}^{5}+3{m}^{2}{x}^{5}{y}^{6}+(m+n-3){x}^{6}{y}^{6}.\end{array}$$

Then

20 $$\begin{array}{rcl}{D}_{x}\,f(x,y) & = & 12m{x}^{4}{y}^{4}+8m{x}^{4}{y}^{5}+16m{x}^{4}{y}^{6}+\frac{5m}{2}(3n-11){x}^{5}{y}^{5}\\  &  & +15{m}^{2}{x}^{5}{y}^{6}+6(m+n-3){x}^{6}{y}^{6},\end{array}$$

21 $$\begin{array}{rcl}{D}_{y}f(x,y) & = & 12m{x}^{4}{y}^{4}+10m{x}^{4}{y}^{5}+24m{x}^{4}{y}^{6}\\  &  & +\frac{5m}{2}(3n-11){x}^{5}{y}^{5}+18{m}^{2}{x}^{5}{y}^{6}+6(m+n-3){x}^{6}{y}^{6},\end{array}$$

22 $$\begin{array}{rcl}{D}_{y}{D}_{x}\,f(x,y) & = & 48m{x}^{4}{y}^{4}+40m{x}^{4}{y}^{5}+96m{x}^{4}{y}^{6}\\  &  & +\frac{25m}{2}(3n-11){x}^{5}{y}^{5}+90{m}^{2}{x}^{5}{y}^{6}+36(m+n-3){x}^{6}{y}^{6},\end{array}$$

23 $$\begin{array}{rcl}{S}_{x}{S}_{y}(f(x,y)) & = & \frac{3}{16}m{x}^{4}{y}^{4}+\frac{1}{10}m{x}^{4}{y}^{5}+\frac{1}{6}m{x}^{4}{y}^{6}\\  &  & +\frac{m}{50}(3n-11){x}^{5}{y}^{5}+\frac{1}{10}{m}^{2}{x}^{5}{y}^{6}+\frac{1}{36}(m+n-3){x}^{6}{y}^{6},\end{array}$$

24 $$\begin{array}{rcl}{{D}_{x}}^{\alpha }{{D}_{y}}^{\alpha }(f(x,y)) & = & {4}^{2\alpha }3m{x}^{4}{y}^{4}+{4}^{\alpha }{5}^{\alpha }2m{x}^{4}{y}^{5}\\  &  & +{4}^{\alpha +1}{6}^{\alpha }m{x}^{4}{y}^{6}+\frac{{5}^{2\alpha }m}{2}(3n-11){x}^{5}{y}^{5}\\  &  & +{5}^{\alpha }{6}^{\alpha }3{m}^{2}{x}^{5}{y}^{6}+{6}^{2\alpha }(m+n-3){x}^{6}{y}^{6},\end{array}$$

25 $$\begin{array}{rcl}{{S}_{x}}^{\alpha }{{S}_{y}}^{\alpha }(f(x,y)) & = & \frac{3}{{4}^{2\alpha }}m{x}^{4}{y}^{4}+\frac{2}{{5}^{\alpha }{4}^{\alpha }}m{x}^{4}{y}^{5}\\  &  & +\frac{4}{{4}^{\alpha }{6}^{\alpha }}m{x}^{4}{y}^{6}+\frac{m}{{5}^{2\alpha }2}(3n-11){x}^{5}{y}^{5}\\  &  & +\frac{3}{{5}^{\alpha }{6}^{\alpha }}{m}^{2}{x}^{5}{y}^{6}+\frac{1}{{6}^{2\alpha }}(m+n-3){x}^{6}{y}^{6},\end{array}$$

26 $$\begin{array}{rcl}{S}_{y}{D}_{x}(f(x,y)) & = & 3m{x}^{4}{y}^{4}+\frac{8}{5}m{x}^{4}{y}^{5}+\frac{8}{3}m{x}^{4}{y}^{6}\\  &  & +\frac{m}{2}(3n-11){x}^{5}{y}^{5}+\frac{5}{2}{m}^{2}{x}^{5}{y}^{6}+(m+n-3){x}^{6}{y}^{6},\end{array}$$

27 $$\begin{array}{rcl}{S}_{x}{D}_{y}(f(x,y)) & = & 3m{x}^{4}{y}^{4}+\frac{5}{2}m{x}^{4}{y}^{5}+6m{x}^{4}{y}^{6}\\  &  & +\frac{m}{2}(3n-11){x}^{5}{y}^{5}+\frac{18}{5}{m}^{2}{x}^{5}{y}^{6}+(m+n-3){x}^{6}{y}^{6}\end{array}$$

28 $${S}_{x}Jf(x,y)=\frac{3}{8}m{x}^{8}+\frac{2}{9}m{x}^{9}+\frac{2}{5}m{x}^{10}+\frac{m}{20}(3n-11){x}^{10}+\frac{3}{11}{m}^{2}{x}^{11}+\frac{1}{12}(m+n-3){x}^{12},$$

29 $$\begin{array}{rcl}{S}_{x}J{D}_{x}{D}_{y}f(x,y) & = & \frac{48}{8}m{x}^{8}+\frac{40}{9}m{x}^{9}+\frac{96}{10}m{x}^{10}\\  &  & +\frac{25m}{20}(3n-11){x}^{10}+\frac{90}{11}{m}^{2}{x}^{11}+3(m+n-3){x}^{12},\end{array}$$

30 $$\begin{array}{rcl}{{S}_{x}}^{3}{Q}_{-2}J{{D}_{x}}^{3}{{D}_{y}}^{3}f(x,y) & = & \frac{{\rm{512}}}{{\rm{9}}}m{x}^{6}+\frac{{\rm{16000}}}{{\rm{343}}}m{x}^{7}\\  &  & +{\rm{108}}m{x}^{8}+\frac{{\rm{15625}}m}{{\rm{1024}}}(3n-11){x}^{8}\\  &  & +\frac{{\rm{1000}}}{9}{m}^{2}{x}^{9}+\frac{{\rm{5832}}}{{\rm{125}}}(m+n-3){x}^{10}.\end{array}$$

1. First Zagreb Index
  31 $${M}_{1}(G)={({D}_{x}+{D}_{y})f(x,y)|}_{x=y=1}=94m+5m(3n-11)+33{m}^{2}+12n-36.$$
2. Second Zagreb Index
  32 $${M}_{2}(G)={{D}_{y}{D}_{x}(f(x,y))|}_{x=y=1}=220m+\frac{25}{2}m(3n-11)+90{m}^{2}+36n-108.$$
3. Modified Second Zagreb Index
  33 $${}^{m}M_{2}(G)={{S}_{x}{S}_{y}(f(x,y))|}_{x=y=1}=\frac{1}{10}{m}^{2}+(\frac{943}{3600}+\frac{3}{50}n)m+\frac{1}{36}n-\frac{1}{12}.$$
4. Generalized Randic’ Index
  34 $$\begin{array}{rcl}{R}_{\alpha }(G) & = & {{D}_{x}^{\alpha }{D}_{y}^{\alpha }(f(x,y))|}_{x=y=1}=3\times {16}^{\alpha }m+2m{20}^{\alpha }+4m{24}^{\alpha }\\  &  & +\frac{1}{2}{25}^{\alpha }m(3n-11)+3{m}^{2}{30}^{\alpha }+{36}^{\alpha }(m+n-3).\end{array}$$
5. Inverse Randic’ Index
  35 $$\begin{array}{rcl}R{R}_{\alpha }(G) & = & {{S}_{x}^{\alpha }{S}_{y}^{\alpha }(f(x,y))|}_{x=y=1}=\frac{3}{{16}^{\alpha }}m+\frac{2}{{20}^{\alpha }}m\\  &  & +\frac{4}{{24}^{\alpha }}m+\frac{1}{2\times {25}^{\alpha }}m(3n-11)+\frac{3}{{30}^{\alpha }}{m}^{2}+\frac{1}{{36}^{\alpha }}(m+n-3).\end{array}$$
6. Symmetric Division Index
  36 $$SSD(G)={({S}_{y}{D}_{x}+{S}_{x}{D}_{y})(f(x,y))|}_{x=y=1}=\frac{623}{30}m+m(3n-11)+\frac{61}{10}{m}^{2}+2n-6.$$
7. Harmonic Index
  37 $$H(G)=2{S}_{x}J(f(x,y)){|}_{x=1}=\frac{6}{11}{m}^{2}+(\frac{191}{180}+\frac{3}{10}n)m+\frac{1}{6}n-\frac{1}{2}.$$
8. Inverse Sum Index
  38 $$I(G)={S}_{x}J{D}_{x}{D}_{y}{(f(x,y))}_{x=1}=\frac{90}{11}{m}^{2}+(\frac{1673}{180}+\frac{15}{4}n)m+3n-9.$$
9. Augmented Zagreb Index

39 $$\begin{array}{rcl}A(G) & = & {{{S}_{x}}^{3}{Q}_{-2}J{{D}_{x}}^{3}{{D}_{y}}^{3}(f(x,y))|}_{x=1}=\frac{1000}{9}{m}^{2}\\  &  & +(\frac{35698735591}{395136000}+\frac{46875}{1024}n)m+\frac{5832}{125}n-\frac{17496}{125}.\end{array}$$

## Conclusions and Discussion

In the present article, we computed closed form of M-polynomial for *α*-boron nanotubes and then we derived many degree-based topological indices as well. Some other degree based topological indices of boron nanotubes are given in^39^. Topological indices thus calculated for these nanotubes can help us to understand the physical features, chemical reactivity, and biological activities. In this point of view, a topological index can be regarded as a score function which maps each molecular structure to a real number and is used as descriptors of the molecule under testing. These results can also play a vital part in the determination of the significance of ∝- boron nanotubes in electronics and industry. We also want to remark that a thorough comparison of *α*-boron nanotubes can be made with triangular boron Nanotubes^11^. For the rest of this article we reserve symbol *T* for Triangular boron tube and *P* for *α*-boron nanotube.

We give a detailed comparative analysis of degree-based topological indices of both boron tubes. It has been experimentally verified that the first Zagreb index is directly related with total π -electron energy of the structure^33,40^ and references therein. So structure having high values of First Zagreb Index have higher total π -electron energy. From the following Fig. 6 it is evident that total π -electron energy of alpha-Boron nanotube is less than triangular Boron tubes for *m* ≤ 9 and for *m* ≥ 10, total π -electron energy of alpha-Boron nanotube rises sharply as compared to triangular Boron tubes with increase in m.

**Figure 7:**
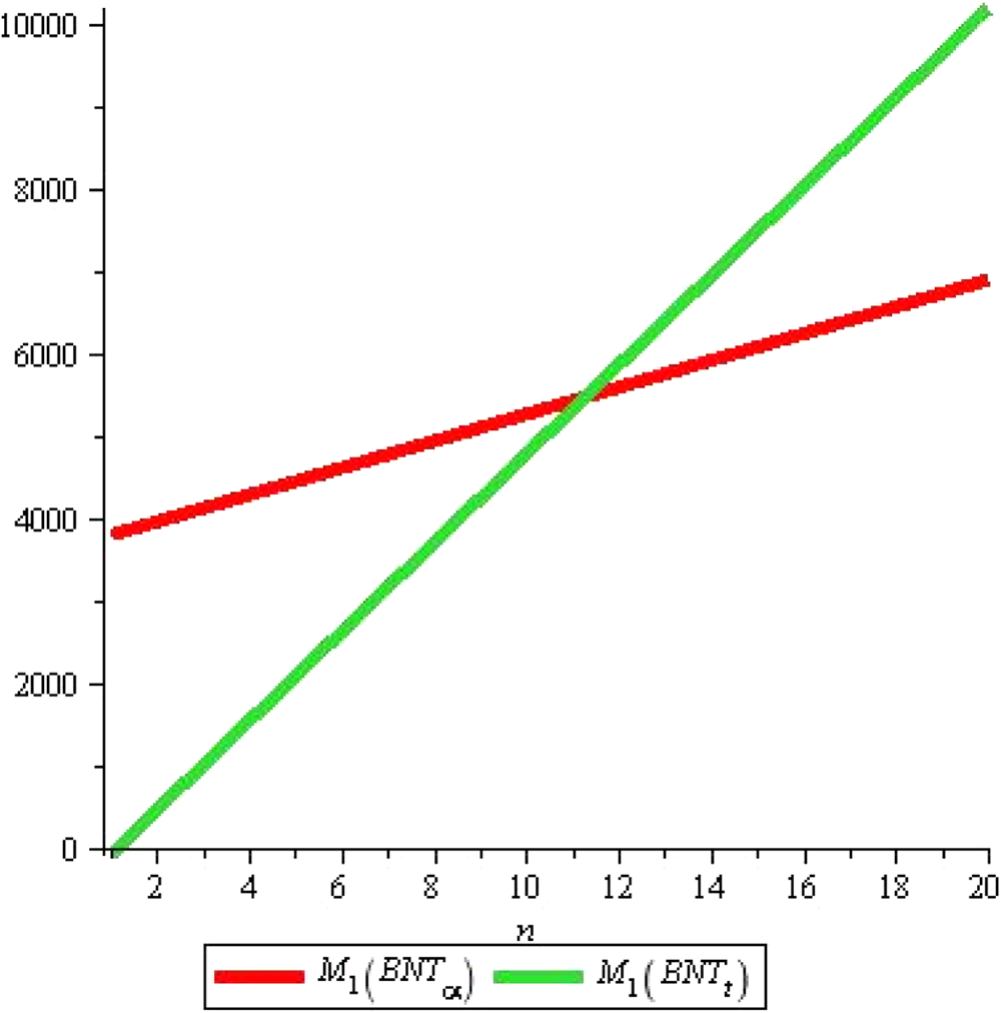
Plot of the first Zagreb index.

**Figure 8:**
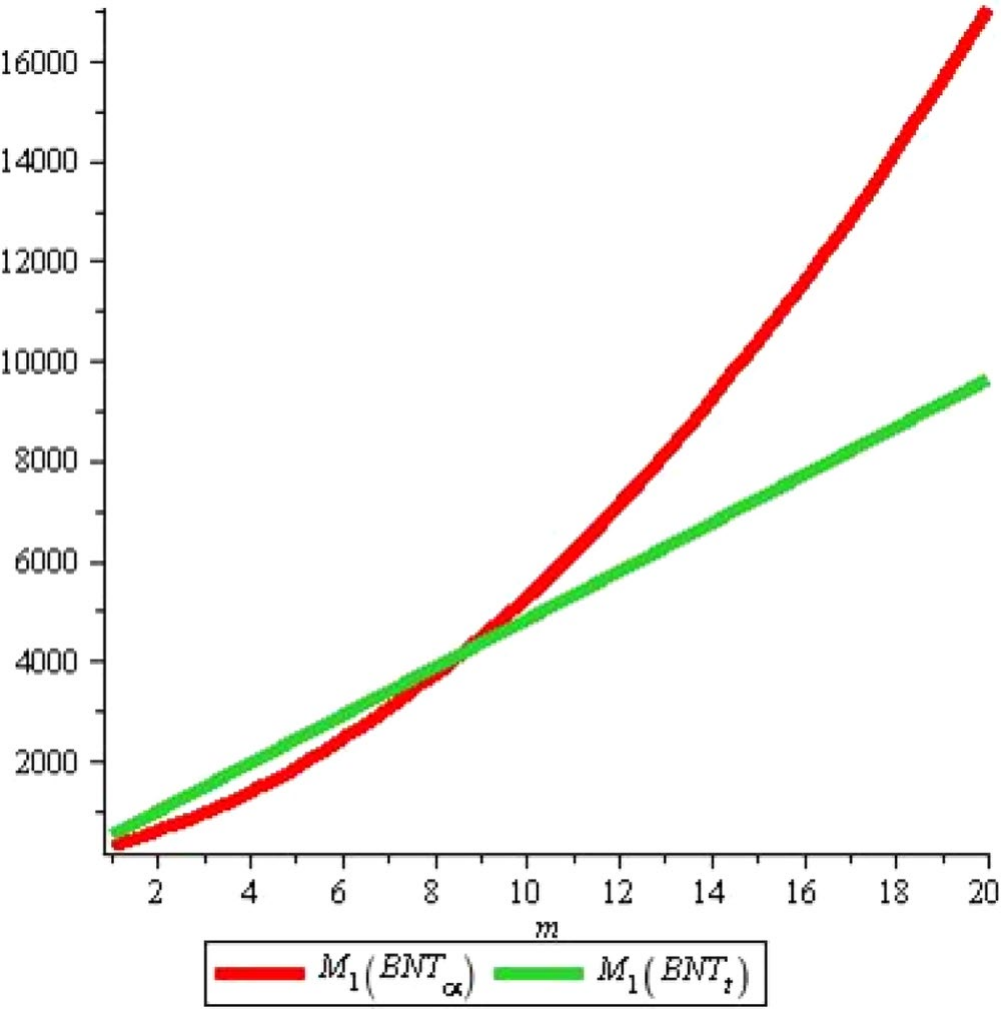
Plot of the First Zagreb index.

Similarly the given Figs 7 and 8 elaborates that total π -electron energy of alpha-Boron nanotube is larger than triangular Boron tubes for *n* ≤ 11 and for *n* ≥ 11, total π -electron energy of triangular Boron tubes rises sharply as compared to alpha Boron tubes with increase in *n*.

**Figure 9:**
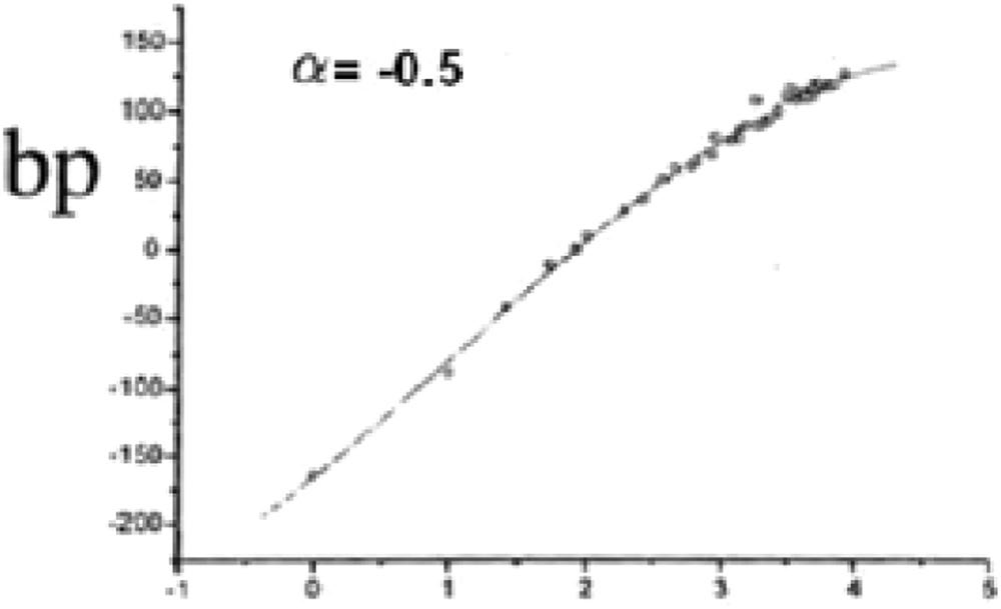
Correlation of the Randic index with the boiling point of a selected set of Alkanes.

Similarly Randic index is useful for determining physio-chemical properties of alkanes as noticed by chemist Melan Randic in 1975. He noticed the correlation between the Randic index R and several physico–chemical properties of alkanes like, enthalpies of formation, boiling points, chromatographic retention times, vapor pressure and surface areas. Following Fig. 9 is adapted from^21^ relating to boiling point of some Alkanes and its correlation with Randic index.

**Figure 10:**
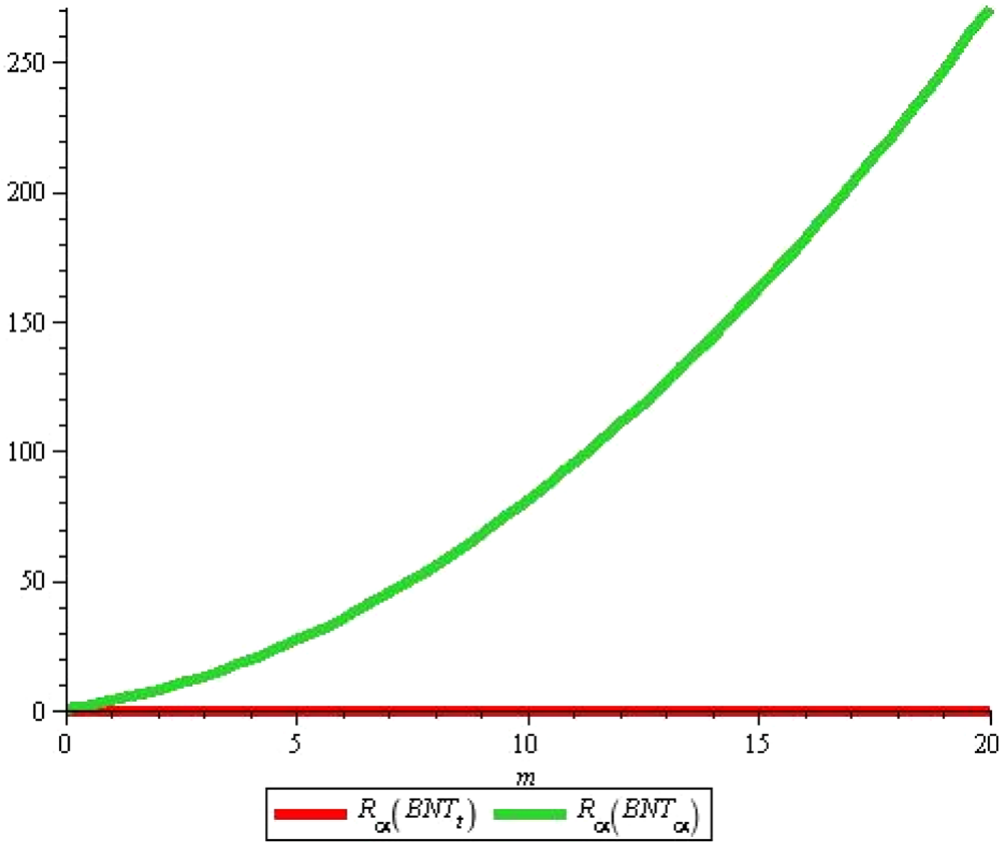
Figure for comparison of *R*_*α*_(*BNT*_*t*_) and *R*_*α*_(*BNT*_*α*_) for *n* = 10 and $m=0\ldots 20$.

Now we give some comparative remarks showing some correlations. The next Fig. 10 clearly depicts that green color rises sharply as compared with red indicating that alpha tube have significant correlation coefficients of above said properties over the triangular boron tubes with the rise in *n* and *m*.

**Figure 11:**
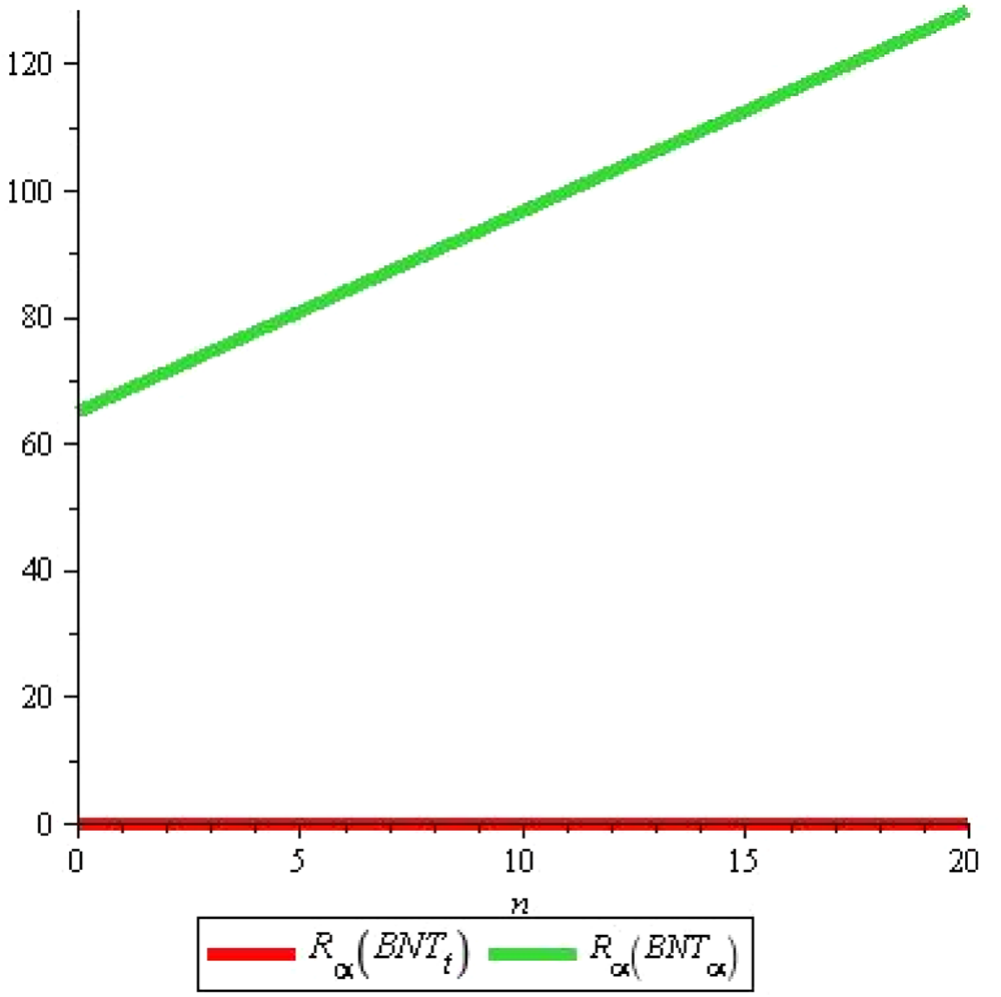
Figure for comparison of *R*_*α*_(*BNT*_*t*_) and *R*_*α*_(*BNT*_*α*_) for *m* = 10 and $n=0\ldots 20$.

**Figure 12:**
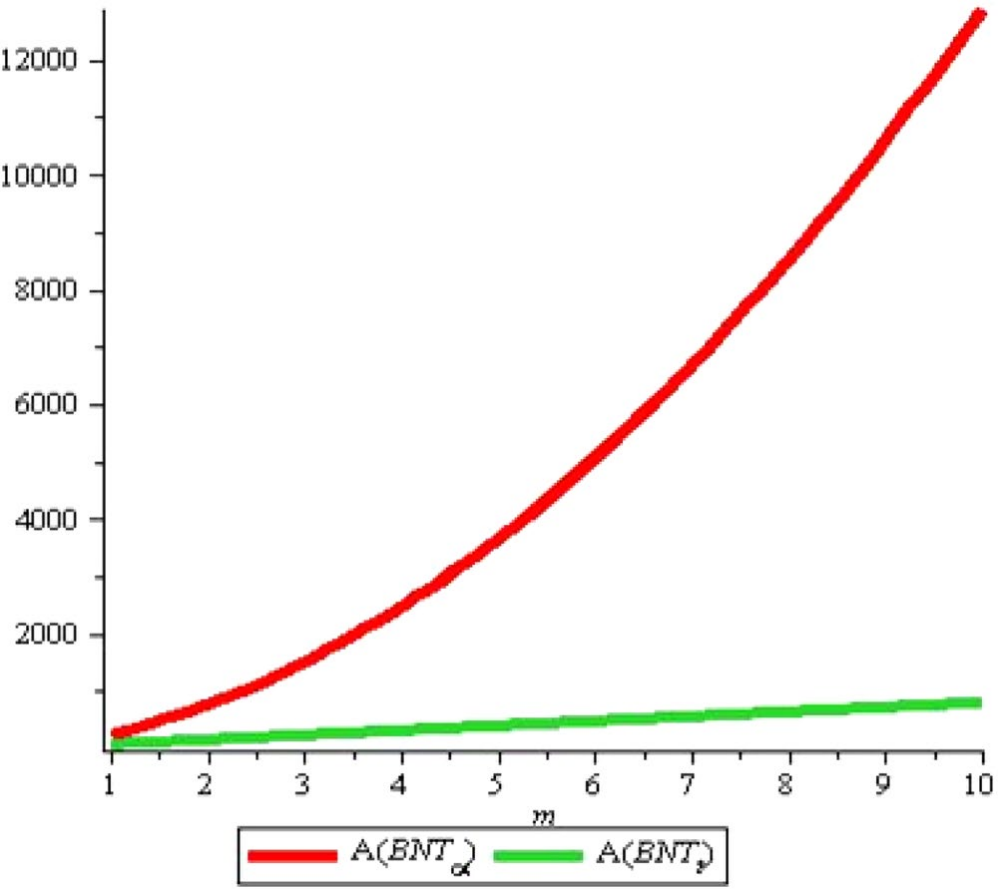
*A*(*G*) for *n* = 2, *m* = *1* … *10*.

**Figure 13:**
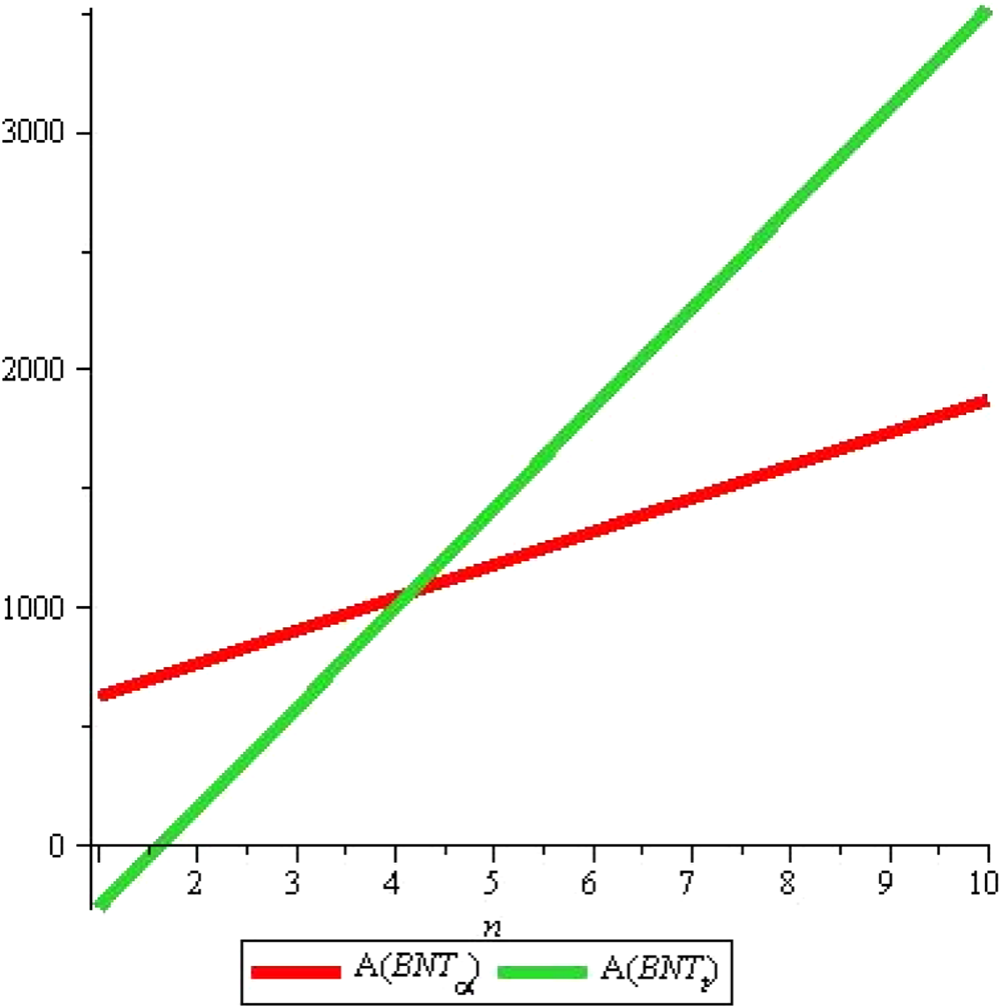
*A*(*G*) for *m* = 2, *n* = *1* … *10*.

It is noticeable from above Fig. 11 that boiling point and other above properties are correlated with Randic index. Subsequent years of research showed that Randic index has a variety of applications specially in medicinal and pharmacological issues. For the results about Triangular boron, we refer to^11^. We give comparative analysis of both tubes for Augmented Zagreb index, Inverse sum index and Harmonic index denoted by *A*, *I* and H respectively. Remaining can be traced out in similarly. We start with *A*(*G*), the Augmented Zagreb index. Recently it has been proved that this index has relatively high correlation coefficient so it can be used for designing quantitative structure-property relations. Figures 12 and 13 shows the graphs of *A*(*G*) for both tubes. We use red color for graphs of *α*-boron nanotubes whereas blue is used for triangular boron tubes. Figure 12 shows that *A*(*G*) decrease linearly with the rise in *m* for Triangular boron whereas it rises sharply for *α*-boron nanotubes. One who wishes to select a boron tube with high *A*(*G*) should naturally choose *α*-boron nanotubes. Figure 13 shows that if we fix m instead of *n*, *A*(*G*) rises linearly with both types of tube although for *n* < 4, *A*(*T*) < *A*(*P*) and *A*(*T*) > *A*(*P*) for *n* > 4. Critical fact is the value *n* = 4 where *A*(*T*) = *A*(*P*). These figures also suggest the length and width of tubes for the desired values of *A*(*G*).

Now we discuss the Inverse sum index.

**Figure 14:**
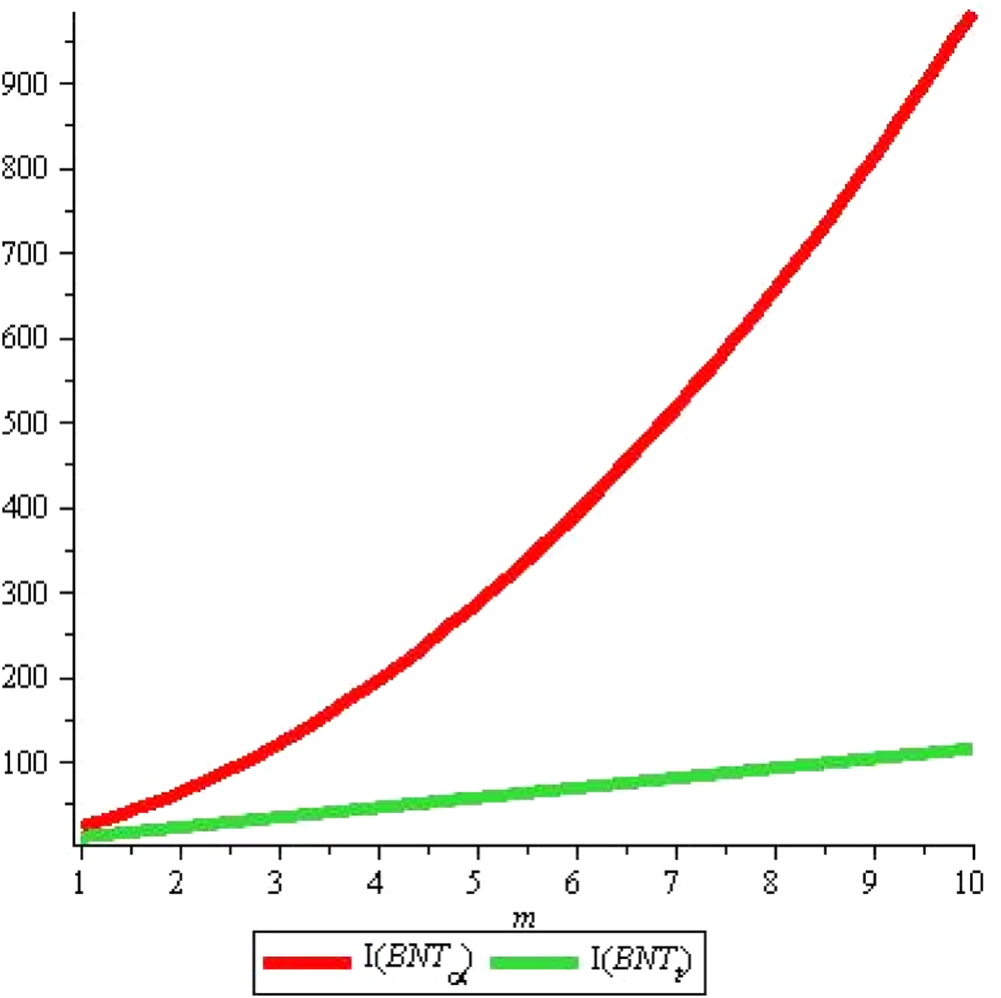
*I*(*G*) for *n* = 2, *m* = *1* … *10*.

**Figure 15:**
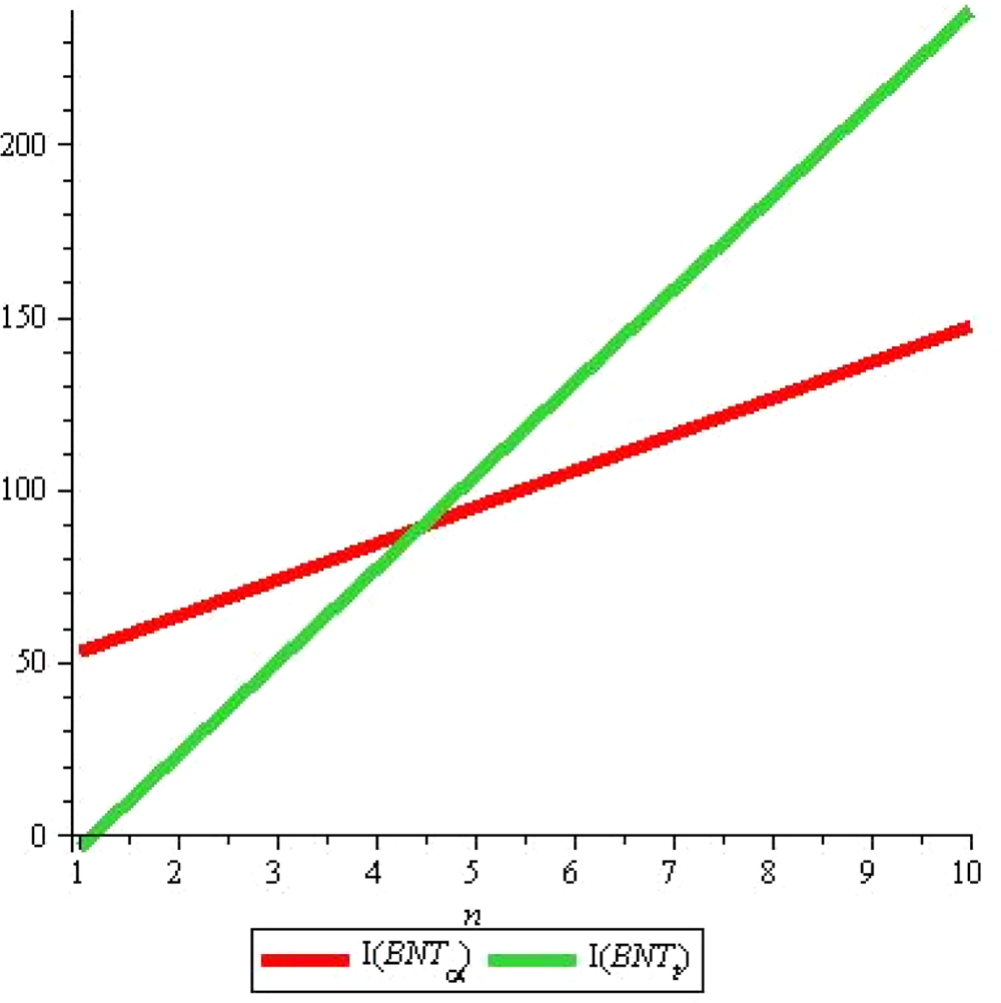
*I*(*G*) for *m* = 2, *n* = *1* … *10*.

As the Fig. 14 suggests that *I*(*P*) rises sharply with the rise in *m* but *I*(*T*) slopes downward very slowly with the same rise if we fix *n* = 2. Whereas both *I*(*P*) and *I*(*T*) rise linearly with rise in *n* although rise in *I*(*T*) seems to be negligible see Fig. 15.

**Figure 16:**
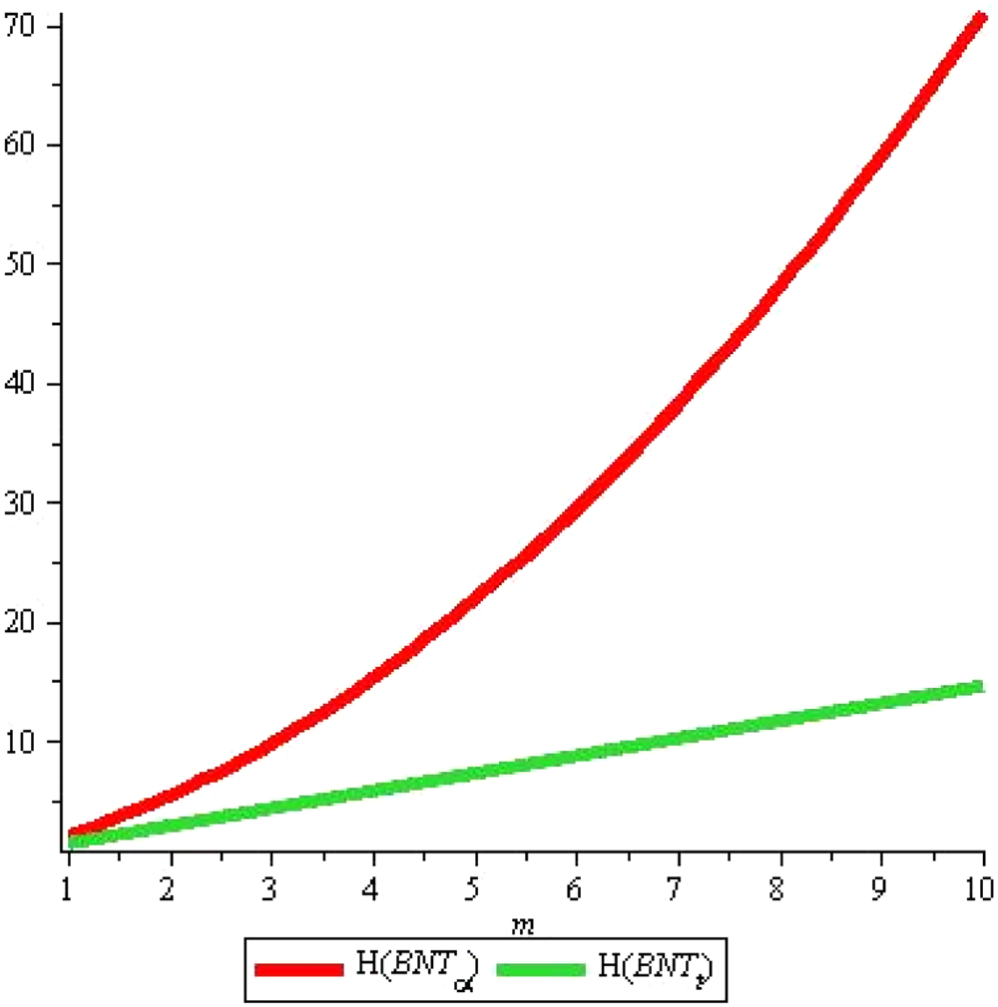
*H*(*G*) for *n* = 2, *m* = *1* … *10*.

**Figure 17:**
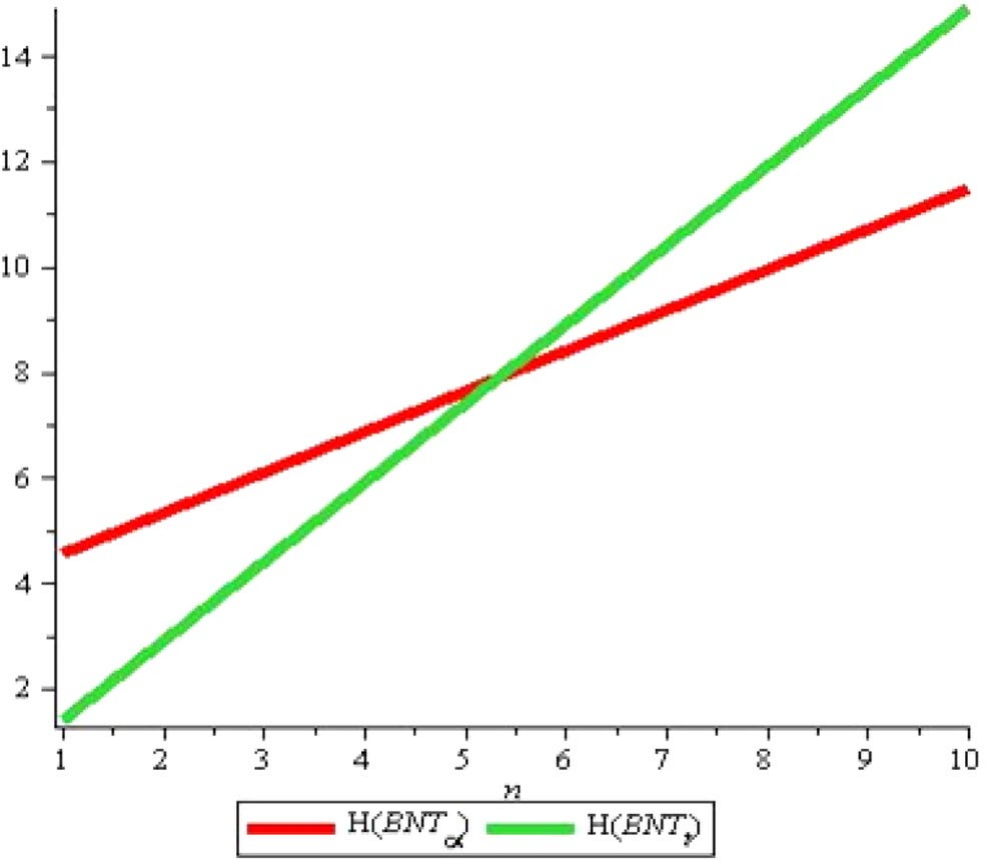
*H*(*G*) for *m* = 2, *n* = *1* … *10*.

For *H*(*G*), we can see that again *H*(*P*) ≥ *H*(*T*) for all positive values of m with *n* = 2, see Fig. 16. Fixing *m* = 2, we can see that for *n* > 3, *H*(*P*) ≥ *H*(*T*) and *H*(*P*) < *H*(*T*) for *n* > 3 but for *n* = 3, we get *H*(*P*) = *H*(*T*), see Fig. 17. We believe that all above computed indices show more or less similar results, so in the end we conclude that *α*-boron nanotubes are far better in obtaining higher values of degree-based topological indices than triangular boron nanotubes.

Above graphs of topological indices show the correlation of *M*_1_(*G*), *R*_*α*_(*G*), *A*(*G*), *I*(*G*) and *H*(*G*) with *m* and *n*. It is clear that all topological indices have linear relation with n whereas graphs are parabolas in relation to m. These facts give an insight idea to control topological indices with length and width of these tubes. Moreover we can find extreme values of topological indices for some definite values of *m* and *n*. We give structural analysis for only three indices as all other indices discussed above show similar trends. We also conclude that *α*-Boron nanotubes have high correlation coefficient regarding Randic index^41–44^.
